# PureCN: copy number calling and SNV classification using targeted short read sequencing

**DOI:** 10.1186/s13029-016-0060-z

**Published:** 2016-12-15

**Authors:** Markus Riester, Angad P. Singh, A. Rose Brannon, Kun Yu, Catarina D. Campbell, Derek Y. Chiang, Michael P. Morrissey

**Affiliations:** Novartis Institutes for BioMedical Research, Cambridge, MA USA

**Keywords:** Purity, Ploidy, Heterogeneity, Whole exome sequencing, Hybrid capture, Copy number, Loss of heterozygosity, Cell lines

## Abstract

**Background:**

Matched sequencing of both tumor and normal tissue is routinely used to classify variants of uncertain significance (VUS) into somatic vs. germline. However, assays used in molecular diagnostics focus on known somatic alterations in cancer genes and often only sequence tumors. Therefore, an algorithm that reliably classifies variants would be helpful for retrospective exploratory analyses. Contamination of tumor samples with normal cells results in differences in expected allelic fractions of germline and somatic variants, which can be exploited to accurately infer genotypes after adjusting for local copy number. However, existing algorithms for determining tumor purity, ploidy and copy number are not designed for unmatched short read sequencing data.

**Results:**

We describe a methodology and corresponding open source software for estimating tumor purity, copy number, loss of heterozygosity (LOH), and contamination, and for classification of single nucleotide variants (SNVs) by somatic status and clonality. This R package, PureCN, is optimized for targeted short read sequencing data, integrates well with standard somatic variant detection pipelines, and has support for matched and unmatched tumor samples. Accuracy is demonstrated on simulated data and on real whole exome sequencing data.

**Conclusions:**

Our algorithm provides accurate estimates of tumor purity and ploidy, even if matched normal samples are not available. This in turn allows accurate classification of SNVs. The software is provided as open source (Artistic License 2.0) R/Bioconductor package PureCN (http://bioconductor.org/packages/PureCN/).

**Electronic supplementary material:**

The online version of this article (doi:10.1186/s13029-016-0060-z) contains supplementary material, which is available to authorized users.

## Background

Accurate knowledge of tumor purity and copy number is required to understand allelic fractions (the ratios of non-reference to total sequencing reads) of genomic alterations, in particular for determining the clonality of alterations, for somatic vs. germline labelling in the absence of matched normal samples, and for identifying regions of loss of heterozygosity (LOH). Furthermore, especially in datasets with high variance in tumor purity across samples, adjustment of purity is necessary for accurate calling of copy number alterations.

Existing algorithms are not designed for hybrid capture sequencing data [1–5], do not support samples without matched normal samples [6–11], and/or do not automatically and accurately adjust for tumor purity and ploidy (e.g. [12–15]). Most existing algorithms use copy number data for purity and ploidy estimation and then utilize germline allelic fractions only for the ranking of inferred purity/ploidy solutions, instead of using copy number and allelic fractions of germline and somatic mutations jointly or inference of these values. There is also an unmet demand for methods distinguishing private germline from somatic mutations, and for doing so, algorithms need very accurate estimates of purity and local copy number to achieve acceptable accuracy [16]. Furthermore, for SNV classification, not only total copy number is needed, but also the maternal and paternal copy numbers are important and only few published algorithms for sequencing data provide allele-specific copy numbers (e.g. [3, 8]). A reliable algorithm for classifying private variants would make sequencing of matched normal samples less important, especially in settings such as diagnostics where variants of uncertain significance (VUS) are typically ignored, but where secondary, exploratory analyses are common.

We present a flexible Bioconductor/R package that integrates with (but does not require) standard GATK-based [17] pipelines, utilizes standard Bioconductor infrastructure [18–21] for data import and export, supports both matched and unmatched samples, and was tested on targeted panels. PureCN provides well-tested copy number normalization and segmentation functionality, but can be easily integrated with existing copy number pipelines. While the algorithm builds on existing ideas developed for genome-wide array data, its novel likelihood model was designed and optimized for short read sequencing data with or without matched normal samples. In contrast to existing solutions, this likelihood model identifies artifacts caused by incorrect read alignment or contamination of DNA from other individuals, incorporates the important information provided by somatic point mutations, can use copy number and SNV information jointly, and supports uneven tiling of targets across the genome. PureCN further supports copy number and LOH calling in 100% pure and unmatched samples such as cell lines. Our software is thus widely applicable, both in diagnostic and research settings.

## Implementation

### Data pre-processing

**Fig. 1:**
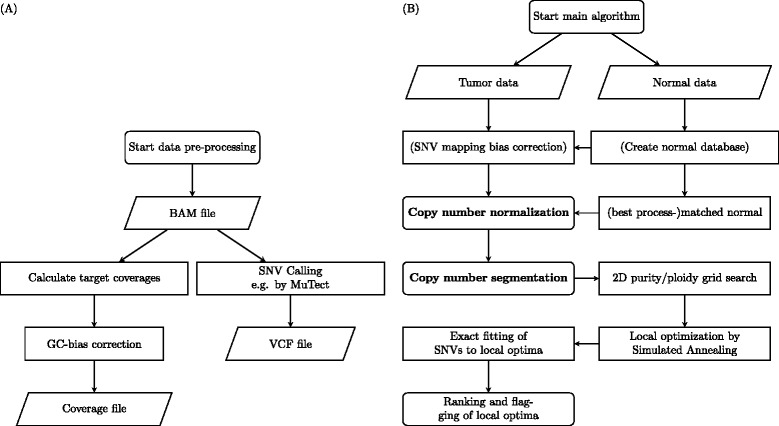
Flowchart of the PureCN data pre-processing pipeline and algorithm. **a** PureCN usually starts from BAM files and calculates average and total coverages for all targeted genomic regions. Coverage data are then corrected for GC-bias. Concurrently, SNVs are called using third-party tools such as MuTect [22]. **b** The main algorithm takes the generated data from tumor as input. If multiple process-matched normal samples are available, the algorithm can optionally use this pool of normal samples to (i) adjust SNV allelic fractions for non-reference mapping bias and (ii) select a best process-matched normal to obtain a clean copy number profile. A pool of normal samples is recommended when matched normal samples are not available. After copy-number normalization and segmentation, local optima for tumor purity and ploidy are obtained via 2D grid search. Integer copy numbers are then assigned to all segments for all local optima via Simulated Annealing. Final likelihood scores are obtained by fitting SNVs to all local optima. If necessary, samples are flagged for manual curation. Steps in *bold font* indicate alternative start points, allowing incorporation of PureCN into third-party copy number pipelines

By default, we start with coverage data calculated from BAM files by either the PureCN calculateBamCoverageByInterval function or by the GATK DepthOfCoverage tool. Both calculate total and average coverages of all targeted genomic regions (Fig. 1a). While it is possible to extract coverage data from germline and somatic single nucleotide variant (SNV) data directly, calculation of coverage across the complete targeted genome utilizes all on-target data and makes the correction of assay-specific capture biases straightforward by utilizing a pool of normal samples. Other biases, most importantly GC bias, are library-specific and should be corrected separately. We thus first GC normalize the coverage data using standard methods [10, 12]. Additionally, SNV data in VCF format are obtained separately using standard third-party tools such as MuTect [22]. All BAM files, from tumor and normal samples, are processed with this pipeline depicted in Fig. 1a. If the tumor and normal samples are matched, then the SNV caller can be run in matched mode to obtain somatic status of variants.

### Copy number normalization and segmentation

Next, for calculating target-level copy number log-ratios, a suitable normal sample is ideally selected from a pool of high quality process-matched normals via principal component analysis (PCA) of GC-normalized coverage data (Fig. 1b). By default, the normal sample with minimum Euclidean distance to the tumor on the first 3 principal components is used for normalization. This procedure selects a normal sample with sufficient coverage and similar library-specific coverage biases compared to the tumor sample. Since coverage is the major source of variance, we scale the coverage for this step to a maximum (defaults to 100×) for samples exceeding this maximum. It is also possible to use the *n* best normals and then provide the normalization function an (weighted) averaged coverage, which is useful when the normal samples are sequenced to significantly lower target coverage than the tumor samples.

Our implementation is modular and allows the incorporation of existing segmentation algorithms. In the default setting, log-ratios of coverage between the tumor sample and PCA-matched normal sample are smoothed and segmented using a weighted version of the circular binary segmentation algorithm implemented in the DNAcopy R package (CBS) (Venkatraman and Olshen, 2007). We set target weights proportional to the inverse of the coverage ratio standard deviations in the pool of normals using the function createTargetWeights. Thus, targets with highly variable coverage in normals, either due to technical artifacts (e.g. mappability issues) or common germline variants, are down-weighted in the segmentation. If no pool of normals is available, the standard, unweighted CBS is used. If a pool of normal samples is available, we further exclude target intervals with low median coverage (by default lower than 20% of the chromosome median).

While heterozygous germline SNPs are sparse, they do provide valuable information for improving segmentations obtained by coverage data only. The number of targets with heterozygous SNPs usually varies between 5 and 15%, depending whether SNPs in flanking regions of targets are included or removed. We find that including SNPs in 50 bp flanking regions add a significant number of high coverage SNPs and we therefore use 50 bp as default, but optimal parameters depend on coverage and assay and should be tuned. Breakpoints of borderline significance (*P* > 0.001) are removed in our default segmentation when mirrored allelic fractions (1- allelic fraction if allelic fraction >0.5) of known heterozygous germline variants (dbSNP) are not significantly different (*P* > 0.2, two-sided *t*-test) in the corresponding neighboring segments. Segments are further tested for copy number neutral LOH. To this end, we recursively identify within all segments the optimal breakpoints that minimize the standard deviations of germline allelic fractions; only if the difference in allelic fraction of the neighboring candidate segments reaches a given alpha threshold, the breakpoint is accepted (two-sided *t*-test). We note that if the assay includes copy number tiling probes highly enriched in heterozygous SNPs, an algorithm (e.g. FACETS or PSCBS [8, 23]) that jointly segments coverage and allelic fractions can sometimes provide better results and we provide a convenient wrapper function for using the PSCBS method over the default. Finally, we use Ward’s hierarchical clustering to find segments with similar copy number log-ratios and mirrored allelic fractions. These segments are normalized to have the same mean log-ratios.

### Purity and ploidy estimation

We first use a 2D grid search to find tumor purity and ploidy combinations that fit the log-ratio profile well. The log-ratios *r*_*i*_ in a segment *i* are assumed to be normally distributed with standard error *σ*_*ri*_, the latter we estimate from the segmentation (i.e., is set to the average standard deviation of log-ratios in a segment). The log-ratios are a function of copy number *C* and purity *p*:

1 $$ {r}_i\sim N\left({ \log}_2\frac{p{C}_i+\left(1-p\right)2}{p\left({\displaystyle {\sum}_j}{l}_j{C}_j\right)/{\displaystyle {\sum}_j}{l}_j+\left(1-p\right)2},{\sigma}_{ri}\right) $$

A difference between our algorithm and most others designed for whole genome data is that segment likelihoods are essentially weighted by the number of exons per segment, not by the base pair segment size *l*. This is an advantage when targets are not evenly distributed, for example in smaller gene panels. The segment size is used for calculating the tumor ploidy in the denominator, using the tumor copy numbers of all *j* segments. Equation (1) assumes that ploidy in normal is 2; PureCN thus detects sex and excludes sex chromosomes for males.

Our algorithm can also take as input already segmented data and *σ*_*ri*_, for example when matched SNP6 data is available. The algorithm will then generate simulated exon-level data given a specified interval file and will use the same likelihood model. Typically, multiple purity/ploidy combinations are equally likely, and we will later use a Bayesian framework to pick the combination that best fits allelic fractions of germline and somatic single nucleotide variants (SNVs), without necessarily requiring knowledge whether these variants are indeed germline or somatic. All local optima identified in the grid search are tested via this framework. This grid search is typically performed in less than 2 min on an average workstation and significantly reduces the search space for the more computationally intensive fitting of variant allelic fractions.

In the grid-search, we assume that all ploidy values are possible, although this is not necessarily true since copy numbers are not continuous. The assumption allows the calculation of the likelihood scores in (1) without knowing the exact integer copy numbers of all segments required in the denominator. Thus every local optimum is in a second step optimized by Simulated Annealing, in which integer copy numbers are assigned to all segments and the purity estimate is fine-tuned. More precisely, Eq. (1) is used to calculate integer copy number posterior probabilities for all segments, *P(C*_*i*_ *)*, and we use a heated Gibbs sampler to optimize the segment copy numbers until convergence, which is, in general, achieved after few iterations. Purity is similarly optimized via heated Gibbs sampling using a specified grid (default from 0.15 to 0.95 in steps of 0.01). We consider copy numbers from 0 to 7 and include a “sub-clonal” state based on a univariate distribution, used for all segments that do not fit integer values and for capturing high-level amplifications with copy number >7 (Carter, et al., [1]).

Mis-calibrated copy number log-ratios (slightly right or left-shifted) can cause shifts in maximum likelihood ploidy estimates when assigning integer copy numbers to segments. In our optimization, we thus re-calibrate the log-ratios by Gibbs sampling. By default, log-ratios are right or left-shifted by at most 0.25 times the mean segment log-ratio standard deviation. If the optimized ploidy is one chromosome higher or lower than the ploidy identified the grid search, additional optimizations are attempted with this re-calibration range increased to up to 1 times the log-ratio standard deviation. The purity/ploidy solution is finally discarded if the optimized ploidy is, after these extensive re-calibrations, still not similar to the grid search ploidy. Mis-calibrations happen when major copy number alterations are not captured and are thus much more frequent in targeted panels without dedicated copy number tiling probes than in whole exome data.

### SNV likelihood model

The next step in our approach is to determine somatic status of SNVs. We fit the allelic fractions of SNVs, provided as VCF file for example generated by the MuTect algorithm [22], to the purity/ploidy combinations of all local optima. We first specify the necessary prior probabilities for SNVs being somatic (vs. germline), *P(g)*. If a matched normal is available, we set it to 0.999 for somatic mutations and 0.0001 for germline variants (note that these do not need to add up to 1, since these priors are assigned to different variants). The reason for not setting these priors to 1 and 0 is to limit the impact of single variants, in particular avoiding rare artifacts dominating the likelihood scores. Without matched normals, we rely on the public databases dbSNP and COSMIC, namely we set the prior to 0.95 if the variant is found more than 2 times in COSMIC; to 0.0005 if the variant found in dbSNP; to 0.01 if found in both COSMIC and dbSNP; and otherwise to 0.5. Accurate calibration of these priors is challenging, since these correspond to error rates in the public databases and these errors are sequence specific, for example errors in COSMIC often cluster in segmental duplications with low coverage and are thus different for different assays. All priors used in the PureCN likelihood model can be tuned by the user. In practice, since the vast majority of variants are germline and present in dbSNP, final results of purity and ploidy are very robust to the choice of these priors.

The expected allelic fraction *f* of variant *i* is a function of tumor purity *p*, copy number *C*, germline status *g* (1 for germline, 0 for somatic) and multiplicity *M*, which is the number of chromosomes harboring the mutation:

2 $$ E\left[{f}_i\right] = \frac{p{M}_i + {g}_i\left(1 - p\right)}{p{C}_i + 2\ \left(1 - p\right)} $$

Note that this does not model homozygous germline variants (*g* is not allowed to be 2), since these are uninformative and are by default removed. Somatic mutations further by definition always have a multiplicity larger than 0 (1 or larger for mono-clonal mutations). We model the sampling variance of allelic fractions using a beta distribution with *n* being the number of covered reads. The likelihood of observing a particular allelic fraction given these parameters is defined as in Carter et al. (Carter, et al., [1]):

3 $$ L\left({f}_i\Big|p,{C}_i,{g}_i,{M}_i,{n}_i\right)= Beta\left(E\left[{f}_i\right]\Big|\ {n}_i{f}_i+1,{n}_i\left(1-{f}_i\right)+1\right) $$

Note that heterozygous germline SNPs with observed allelic fraction significantly different from 0.5 [using (3), *P* < 0.05] in the matched normal or in a sufficient number of samples in the pool of normals are also removed. These are often SNPs in segmental duplications or other low-quality genomic regions. Smaller non-reference biases in regions of high mappability cause only minor shifts in expected allelic fractions and are not explicitly modeled, but we provide functionality to adjust observed allelic fractions, for example by estimating position-specific scaling factors in a large pool of normal samples. With increasing coverage, these biases may lead to very small likelihoods for correct purity and copy number values if not adjusted correctly, causing a paradox where increasing coverage decreases accuracy, and we therefore define a maximum value for *n* (defaults to 300×).

Incorporating the uncertainty of copy number calculated via Eq. (1), (3) becomes:

4 $$ L\left({f}_i\Big|p,{g}_i,\ {M}_i,n\right)={\displaystyle \sum_{C_i\in \left\{0..7\right\}}}P\left({C}_i\right)L\left({f}_i\Big|p,{C}_i,\ g,{M}_i,n\right) $$

We finally integrate over the uncertainty of germline status and multiplicity to find for each variant the most likely state:

5 $$ P\left({g}_i,{M}_i\Big|{f}_i,\ {n}_i\right) = \frac{P\left({M}_i\right)P\left({g}_i\right)L\left({f}_i\Big|p,{g}_i,{M}_i,{n}_i\right)}{{\displaystyle {\sum}_{C_i\in \left\{0..7\right\}}}{\displaystyle {\sum}_{K_i\le {C}_i}}{\displaystyle {\sum}_{M_i\le {C}_i}}{\displaystyle {\sum}_{g_i\in \left\{0,1\right\}}}P\left({C}_{ij}\right)P\left({M}_{ij}\right)P\left({g}_{ij}\right)L\left({f}_i\Big|p,{C}_{ij},{g}_{ij},\ {M}_{ij},{n}_i\right)} $$

Possible values for *M* depend on the number of maternal and paternal chromosomes, with *K* denoting the smaller one of the two chromosome numbers. We assume that the multiplicity of germline variants in a segment correspond to the maternal and paternal chromosome numbers with probability P_K_, by default set to 0.999. By not setting this value to 1, we make the likelihood model more robust to segmentation errors. For somatic mutations, we further always allow the mutation of a single chromosome; this assumes that multiplicities larger than 1 are the result of copy number alterations, almost never of independent mutations resulting in identical base changes. The prior probabilities for *M* are thus:

6 $$ P\left({M}_i\Big|{K}_i,\ {C}_i,\ {g}_i,{P}_K\right)=\left\{\begin{array}{c}\hfill {P}_K\frac{1}{n_s}\kern1.25em  if\ {M}_i = {K}_i \vee \kern0.5em {M}_i = {C}_i-{K}_i\kern0.5em  \vee \left({M}_i\le 1 \wedge {g}_i=0\right)\hfill \\ {}\hfill 0\  if\ {K}_i > \kern1.25em \left\lfloor {C}_i/2\right\rfloor \hfill \\ {}\hfill \begin{array}{cc}\hfill \left(1-{P}_K\right)\frac{1}{C_i+1-{n}_s}\hfill & \hfill otherwise\hfill \end{array}\hfill \end{array}\right. $$

Where *n*_*s*_ denotes the number of utilized “allowed” states covered in the first case of Eq. (6) for a given *K* and *C* combination. This value can range from 1 to 4; in germline SNPs *n*_*s*_ it would be 1 when both maternal and paternal copy numbers are equal and 2 when these two numbers differ. Somatic mutations can have two additional states, the mutation of a single chromosome (when *M* = *1*) and a sub-clonal state (when *M* < *1*). This sub-clonal state is by default modelled in Eq. (5) by replacing the invalid *M* = *0* and *g* = *0* state (somatic mutations by definition have *M* > *0*) with *M* = *1*/*3* and *g* = *0*. This *M* value represents the expected average cellular fraction of sub-clonal mutations.

We assume flat priors for *K*, $P\left({K}_i\right)=\frac{1}{C_i+1}$, but note that databases of samples could provide better priors (see [1] for a related karyotype likelihood model). For example LOH in the *TP53* tumor suppressor is very common in various cancer types; we would thus find the corresponding copy number state *K* = 0 and *C* = 1 frequently in these cancer types. If low ploidy solutions can explain the data well, then this prior further results in favoring low over high ploidy solutions (which is why *K* is defined over the complete copy number range). We however noticed that haploid solutions are often ranked relatively high in low purity samples, because the lack of one tumor chromosome does not result in sufficiently unbalanced germline allelic fractions in those samples. We thus give haploid and diploid solutions the same prior probability when the tumor purity is below 35%. Regions of LOH are classified as LOH or not, using the most likely segment state as determined in Eq. (5); a segment is in LOH if *C* = 1 or *K* = 0.

To model possible contamination from other individuals’ DNA, we optionally include two additional SNV states. The first models homozygous germline SNPs that were not removed because reference alleles were sequenced from the contaminated DNA, resulting in allelic fractions lower than 1 (Eq. 7). The second state (Eq. 8) models SNPs where the non-reference allele is only present in the contamination. The expected allele frequency is now a function of purity, tumor copy number and contamination rate *c*:

7 $$ E\left[{f}_i\right] = \frac{p{C}_i + 2\left(1 - p-c\right)}{p{C}_i + 2\ \left(1 - p\right)} $$

8 $$ E\left[{f}_i\right] = \frac{c}{p{C}_i + 2\ \left(1 - p\right)} $$

The prior probabilities for these states are set to a non-zero value for germline variants (present in dbSNP) only, because the dimension (i.e. number of possible states) for novel variants is much higher, thus rarely resulting in likelihood scores low enough to impact purity/ploidy selection. We set contamination rate and prior probability for this state by default to a low 0.01. The main motivation for this functionality is to provide a bin for germline variants that do not fit any other state, and more specialized tools should be used to detect contamination. We note that if matched normal samples are available, then this step is not crucial, since contamination is identified as non-germline by variant calling algorithms, whereas without matched normal samples, the presence of variants in dbSNP results in high germline prior probabilities.

In samples of 100% purity, homozygous SNPs should not be removed a priori, since these could be heterozygous SNPs in mono-clonal LOH regions. For high purity samples without matched normal samples, we therefore optionally provide yet another germline state, the homozygous state. Any observed reference reads are assumed to be independent sequencing errors resulting in identical base pairs (by default occurring at rate ε = 10^−3^/3) and the state likelihoods are then modeled with a binomial distribution. Flat prior probabilities independent of ploidy are applied.

Finally, for variants most likely being somatic, we calculate the fraction *h* of tumor cells harboring the mutation:

9 $$ h = \left[\frac{f}{M}pC+2\left(1-p\right)\right]\frac{1}{p} $$

The SNV-fit likelihood is the sum of the log-likelihood scores of the most likely states for all variants. The tumor purity/ploidy combinations are finally sorted by sum of the log-likelihood scores of both copy number and SNVs.

Our implementation provides an additional post optimization, in which purity is optimized using both copy number and allelic fractions in the SNV fitting step. This is achieved by adding purity as additional dimension in the denominator of Eq. 5. In default mode, this is turned off, i.e., allelic fractions are only used to select the most likely purity/ploidy combination from the copy number fitting. The accuracy gain for copy number calling is typically marginal in high quality samples with sufficient coverage as used in this benchmarking (data not shown). For classification of variants by somatic status, we recommend turning this feature on as small inaccuracies in purity can decrease the performance significantly since the distributions of allelic fractions of the different SNV states often overlap. By default, we use flat priors for tumor purity, but users can provide priors for all tested purity values in the grid.

### Automated calling

If the algorithm is applied to many samples, it is important to flag samples that likely need manual curation. We flag samples of potentially low quality (noisy segmentations, high AT- or GC-dropout, sample contamination), samples where the maximum likelihood solution has characteristics rarely seen in correct solutions (rare ploidy, excessive LOH, excessive homozygous losses), and samples that are difficult to call (non-aberrant, poly-genomic). We further provide functionality for automatically removing very unlikely optima via a bootstrapping procedure, in which variants are sampled with replacement and optima are then re-ranked. Optima which never rank high in any bootstrap replicate are removed. Bootstrap values may also flag samples for manual curation when PureCN identified multiple plausible solutions. Finally, we calculate for each sample a goodness-of-fit score of the SNV fitting, ranging from 0 to 100%, where 0% corresponds to the worst possible fit and 100% to a perfect fit. We defined the worst possible fit as a fit in which observed allelic fractions differ on average by 0.2 from their expected values. Both low purity and high ploidy solutions are biased towards higher scores; low purity allelic fractions have a low variance in general and high ploidy solutions are complex and usually find good fits. Compared to log-likelihood scores, however, this goodness-of-fit score is intuitive and allows a straightforward flagging of very poor fits.

## Results

### Example

**Fig. 2:**
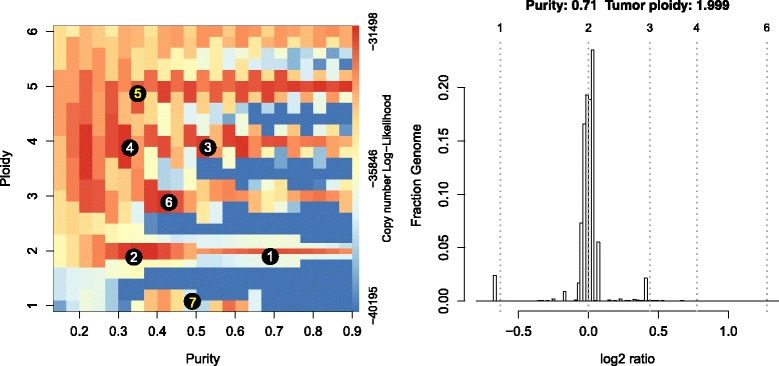
Example output of PureCN, applied to whole exome sequencing data of a male breast cancer patient. The first step in PureCN is fitting exon-level copy-number to all purity and ploidy combinations in a 2D grid search. The colors visualize the copy number fitting log-likelihood score from low (*blue*) to high (*red*). The *numbers* indicate local optima and their final rank after fitting both copy number and allelic fractions of germline SNPs and somatic mutations

We applied our implementation to whole exome sequencing data from a male breast cancer metastasis sample [24]. After segmentation, initial estimates of purity and ploidy were obtained in a grid search, and allelic fractions of SNVs were fitted to all local optima. Figure 2a shows a surface plot, in which likelihood scores were colored from blue (low) to red (high), with the numbers showing the final ranks of all tested local optima after fitting both copy number and allelic fractions. Figure 2b displays a histogram of tumor vs. normal copy number log-ratios for the maximum likelihood solution (number 1 in Fig. 2a). The height of a bar in this plot is proportional to the fraction of the genome falling into the particular log-ratio copy number range. For a given purity and ploidy combination, the vertical dotted lines visualize the expected log-ratios for all relevant integer copy numbers; it can be seen that most of the log-ratios of the maximum likelihood solution align well to expected values for copy numbers of 1, 2 and 3.

**Fig. 3:**
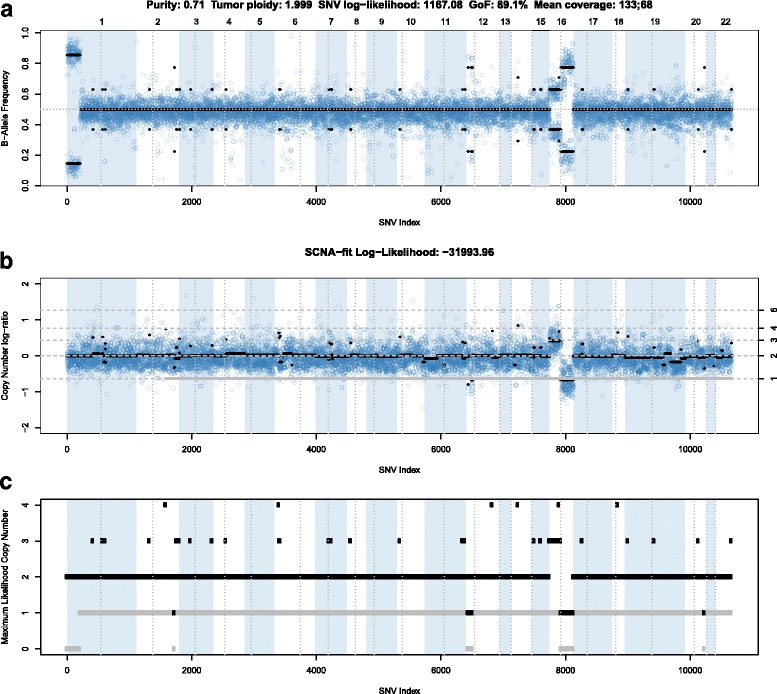
B-allele plot of the male breast cancer metastasis example. In all 3 panels, a *dot* represents a germline SNP (in single sample mode a predicted germline SNP). The first panel (**a**) shows their allelic fractions along the genome. *Background colors* visualize chromosomes and *vertical dotted lines* centromere positions. The *bold black lines* visualize the expected (not the average) allelic fractions in the segment. These expected values are calculated using the estimated purity and the total (sum of maternal and paternal copy number) and minor (the minimum of maternal and paternal copy number) segment copy numbers (Eq. 5). These are visualized in *black* and *grey*, respectively, in the (**b**) and (**c**). Panel (**b**) shows the copy number log-ratios, panel (**c**) the final, inferred integer copy numbers. SNPs plotted as *triangles* or *crosses* were classified as potential homozygous or contamination, respectively

**Fig. 4:**
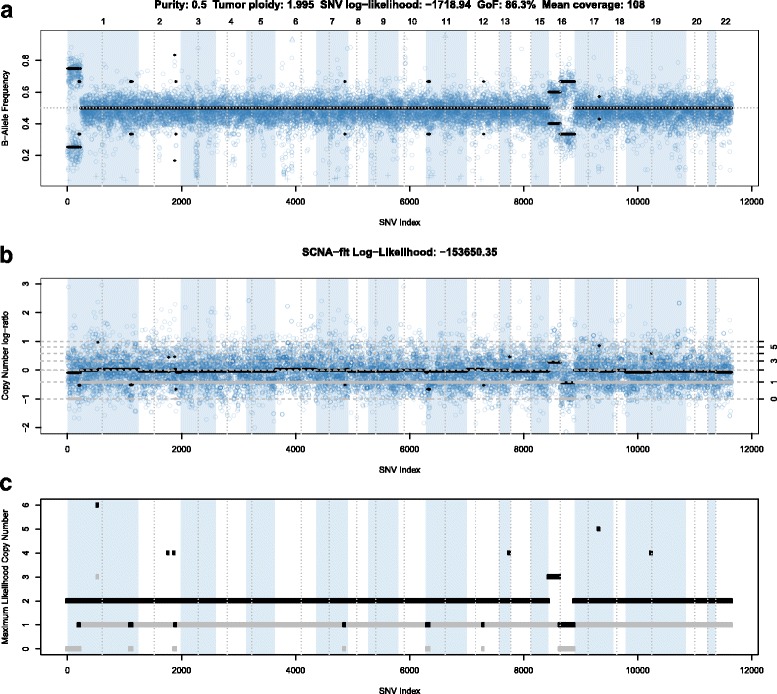
B-allele plot of the male breast cancer primary tumor sample. *Panels* are as described in Fig. 3, but data is for the matched primary tumor run in tumor-only mode, without the matching normal sample. The lower purity results in log-ratios and B-allele frequencies closer to 0 and 0.5, respectively

**Fig. 5:**
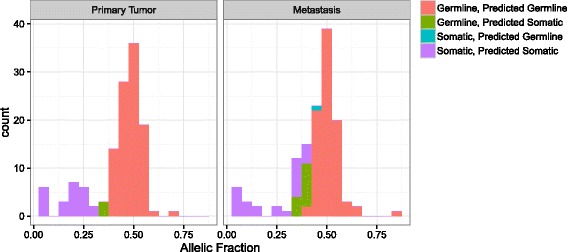
Accuracy of germline vs. somatic variant labeling. This figure displays the allelic fractions of correctly and incorrectly labeled variants for two samples run in MuTect and PureCN without matched normal sample. The ground truth was obtained by running MuTect with matched normal sample. The first was a primary tumor sample with purity of 0.5, the second a metastasis sample with purity of 0.7. Due to the lower purity of the primary tumor sample, the distributions of allelic fraction overlap less, making the prediction easier. The *bars* for the 4 different categories are stacked

Germline variant data are informative for calculating integer copy number, because unbalanced maternal and paternal chromosome numbers in the tumor portion of the sample lead to unbalanced germline allelic fractions (Eq. 2, Fig. 3a). Chromosomes 1p, 12p, 16q, and 16p show deviations from the expected allelic fraction of 0.5 suggestive of structural variants. Figure 3b shows the corresponding copy numbers, first as log-ratios and then as inferred integer copy numbers. Combining these data with the allelic fraction data (Fig. 3a), we find that the LOH of chromosome 1p is copy number neutral, 12p and 16q have LOH due to copy loss and there is a copy number gain of 16p. For this sample, PureCN returned a very similar maximum likelihood purity and ploidy estimate when run with and without the matched normal sample (0.7 for purity and 2.001 for ploidy). When run without matched normal sample, we classified 129 private variants as somatic or germline, and 88.3% were correctly classified. All misclassified variants had allelic fractions between 0.35 and 0.5, the expected fractions for heterozygous somatic and germline, respectively. For the same patient, a primary tumor sample was also available (Fig. 4). The use of a best process-matched normal sample resulted in a clean copy number profile. Some genomic regions display biased allelic fractions of heterozygous germline SNPs, for example the largest one on chromosome 3q, which are automatically removed when matched normal samples are available. In tumor-only mode, these SNPs can be removed by using a pool of normal samples as described in the Method section. The lower tumor purity of 0.5 resulted in a larger difference in expected fractions for somatic and germline variants. With this spread, the accuracy increased to 97.7% (Fig. 5). Most of the private variants exclusive to the either primary tumor or metastatic sample displayed allelic fractions significantly lower than expected for a heterozygous somatic variant, indicating sub-clonality and are labelled such by PureCN [24].

### Benchmarking

**Fig. 6:**
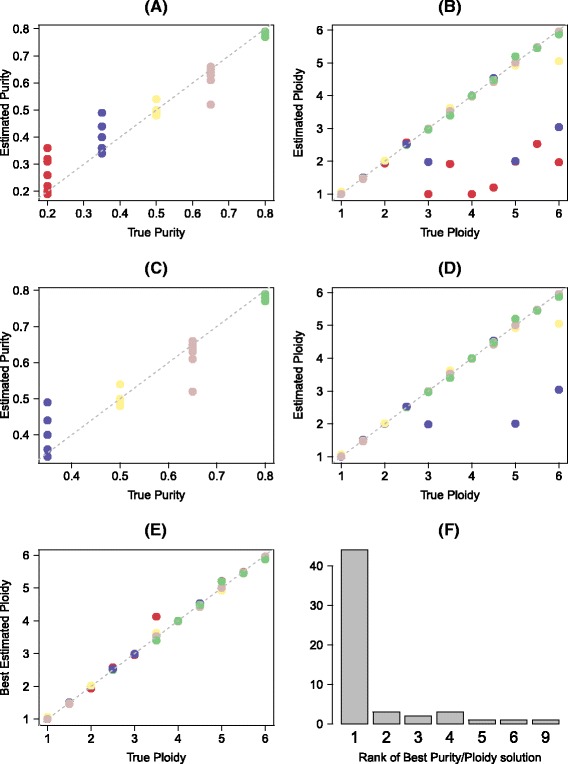
Accuracy on simulated data. The two panels (**a**) and (**b**) show the high correlation of true and inferred maximum likelihood tumor purity and ploidy in simulated data. *Colors* visualize the simulated purity, ranging from 20 to 80%. Excluding the samples with tumor purity of 20%, the correlations increase to 0.98 for purity and 0.95 for ploidy [(**c**) and (**d**)]. Note that for almost all of the samples, the correct solution was considered, but was not always ranked as maximum likelihood solution. Panel (**e**) shows for all samples, including the 20% samples, the ploidy correlation of the best considered solution (minimizing the Euclidean distance in scaled purity and ploidy), for example selected in a hypothetical perfect manual curation (Pearson 0.98). Panel (**f**) shows the histogram of ranks of the best solution over all samples

**Fig. 7:**
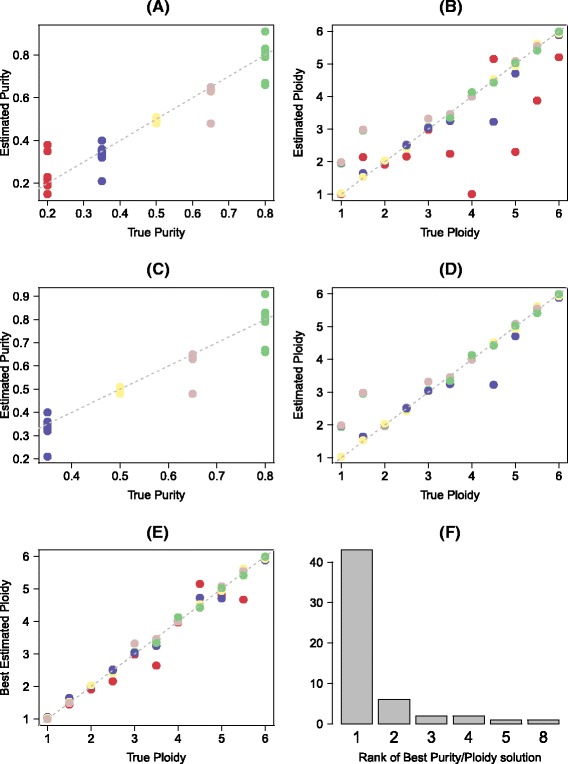
Accuracy on simulated ultra-deep sequencing data (400×) from a 560 gene panel. This shows the same plots as in Fig. 6, but forsimulated data from a targeted panel. Due to the higher sequencing depth, even the low purity samples have in general ploidy estimates close to the true ploidy

We first demonstrated the accuracy of our algorithm on simulated data: artificial whole exome data (100×) and data from an ultra-deep sequenced (400×) 560-gene panel, with purity ranging from 20 to 80%, and ploidy ranging from 1 to 6 (Figs. 6 and 7). Simulated data was based on whole exome and targeted panel data from normal samples. In brief, simulated genomes were first generated by using random segmentations obtained from the TCGA breast cancer study [25] as template. Assuming copy number of 2 for all targets, normal coverage was then scaled to simulated copy number and then scaled to desired target coverage. Allelic fractions were then sampled using Eq. (3), with SNPs randomly assigned to either the maternal or the paternal chromosome. For the whole exome data, the Pearson correlation of true and inferred maximum likelihood purity and ploidy was 0.98 and 0.80, respectively. When excluding samples with purity lower than 35%, the correlations increased to 0.98 for purity and 0.95 for ploidy. For the gene panel, the correlation of purity and ploidy was 0.96 and 0.89, respectively (0.96 and 0.97 excluding low purity samples).

**Fig. 8:**
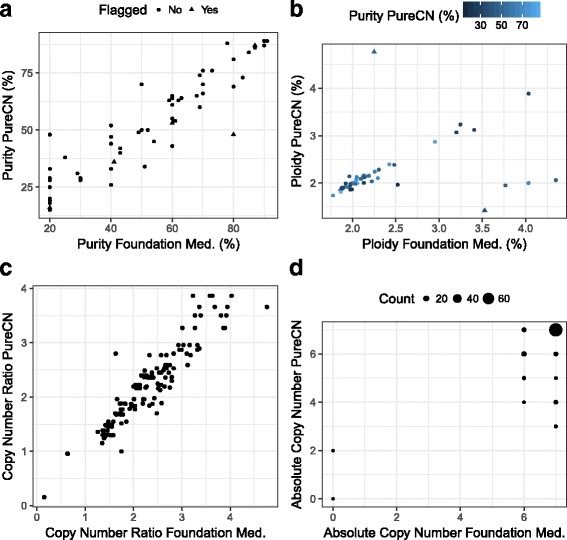
Comparison with existing proprietary ABSOLUTE implementation. In panel (**a**) and (**b**), our purity and ploidy estimates (y-axis), respectively, are plotted against the estimates from Foundation Medicine. *Triangles* indicate solutions flagged by PureCN for manual curation. **c** Compares copy number ratios of amplifications and homozygous deletions in 40 different samples. **d** shows the corresponding absolute copy numbers adjusted for purity and ploidy. Copy numbers were capped at 7 in both algorithms, the default copy number cutoffs for non-focal amplifications

We next applied our algorithm to real sequencing data from 58 cancer samples (Fig. 8), which were obtained from a commercial vendor using a targeted panel with copy number tiling probes [26] and internally using whole exome sequencing on remnant DNA. Libraries were constructed with Illumina TruSeq, captured with the Agilent SureSelect Whole Exome v4 baits, and sequenced on an Illumina HiSeq2500 as 100 base-pair paired-end reads. No matched normal data was available. Foundation Medicine provides purity and ploidy estimates obtained using an unpublished proprietary algorithm that was systematically validated using cell line mixture experiments [26]. To our knowledge, this is the only other algorithm validated for unmatched targeted sequencing data.

We observed a Pearson correlation of 0.92 when comparing our maximum likelihood purity estimate with the estimate provided from Foundation Medicine. Comparing estimated ploidy values, we found that 89.7% of samples showed concordant ploidy estimates (since the vast majority of samples were diploid, the low Pearson correlation of 0.38 is driven by the 10.3% discordant outliers). Discordant samples were mainly of low quality (low purity and one sample with high AT-dropout). Four samples were flagged for manual curation, including two of the three samples for which the PureCN maximum likelihood ploidy estimate was wrong (Fig. 8a and b). These two samples are shown in Additional file 1. Additionally, since copy number fitting involves a Simulated Annealing optimization for assigning integer copy number to segments, we examined the number of iterations until convergence (Additional file 2). Convergence was usually achieved before iteration 20, and we found no correlation of number of iterations and purity or ploidy.

We compared copy numbers of all amplifications and homozygous deletions called by Foundation Medicine and found a good concordance of copy numbers un-adjusted (Fig. 8c) and adjusted (Fig. 8d) for purity and ploidy. Forty samples had at least one called amplification or deletion. The Pearson correlation of un-adjusted copy number ratios was 0.93 (Fig. 8c). For 85% of the samples, the mean difference in absolute copy numbers was within ±1 when comparing PureCN with the Foundation Medicine calls (Fig. 8d).

## Limitations

PureCN was designed for high-coverage (>100×) targeted sequenced data. Quality of results obtained from lower coverage data depends on tumor purity and evenness of coverage. Furthermore, due to increasing sampling variance of allelic fractions, accurate classification of SNVs becomes challenging with decreasing coverage. The automatic classification of all SNVs further results in longer runtimes than other purity/ploidy inference tools (whole-exome runtime for PureCN 1.6 is about an hour without post-optimization), currently practically prohibiting the use of PureCN for whole-genome data. Future versions might see runtime improvements due to implementations of heuristics that eliminate unlikely local optima early.

## Conclusions

PureCN is a flexible open source R/Bioconductor package that assists in understanding allelic fractions of SNVs. Since purity adjusted copy number is important to this end, PureCN is also a state of the art copy number caller for hybrid capture sequencing data, supporting tumor samples with or without matching normal samples.

## Additional files

Additional file 1: Whole-exome samples with wrong maximum likelihood solutions. (DOCX 604 kb)
Additional file 2: Number of Simulated Annealing iterations. (PDF 5 kb)

## Abbreviations

LOH: Loss of heterozygosity
SNV: Single nucleotide variants
VUS: Variants of uncertain significance
