# Supplementary material for: PureCN: copy number calling and SNV classification using targeted short read sequencing
Source: Source Code Biol Med. 2016 Dec 15;11:13. doi: 10.1186/s13029-016-0060-z (PMC5157099; doi:10.1186/s13029-016-0060-z)

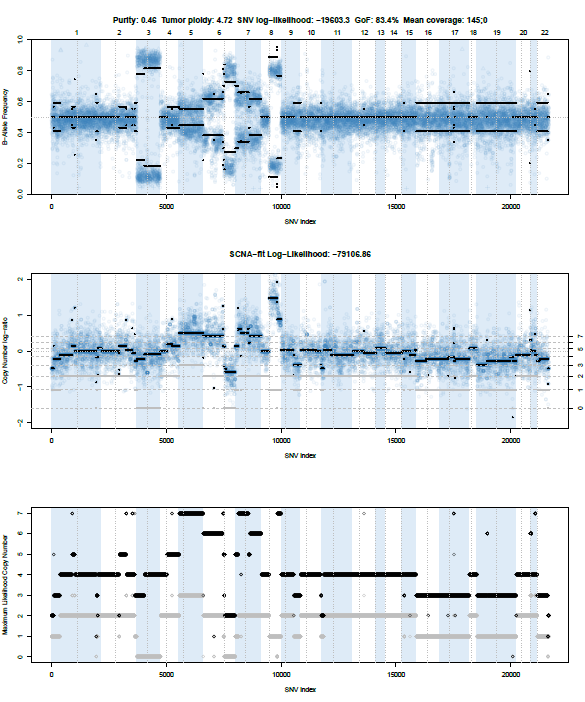


High AT-dropout in this sample resulted in high variance of log-ratios. PureCN with default parameters failed to normalize the coverage properly. No good solution was identified for this sample and this sample thus failed PureCN calling.


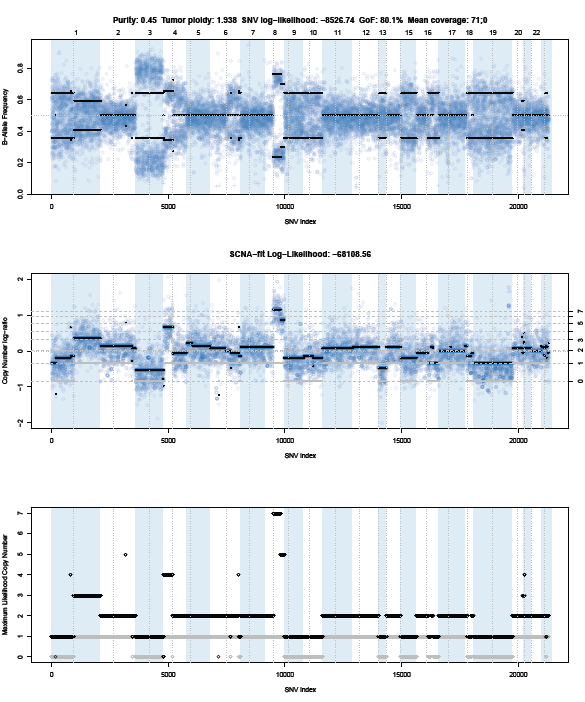


This sample had relatively low coverage of 71X and most chromosomes were consistent with a diploid genome. The second best solution shown on the next page however fits all chromosomes very well. An alternative explanation would be that this is a poly-genomic sample, with the single copy number losses all occurring in a sub-clone. One additional sample was similarly misclassified, with a low-ploidy solution explaining most of the genome very well.


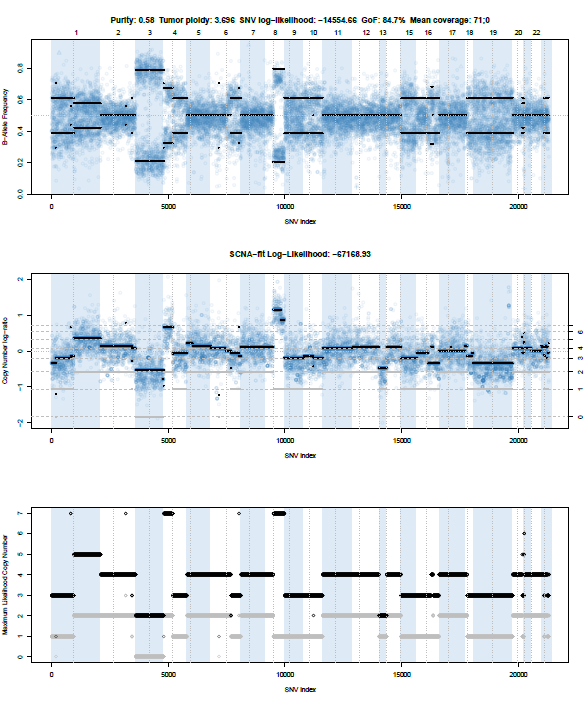

Supplement: Additional file 1: — Whole-exome samples with wrong maximum likelihood solutions. (DOCX 604 kb) [file 13029_2016_60_MOESM1_ESM.docx]
