# Supplementary figures and images for: PureCN: copy number calling and SNV classification using targeted short read sequencing
Source: Source Code Biol Med. 2016 Dec 15;11:13. doi: 10.1186/s13029-016-0060-z (PMC5157099; doi:10.1186/s13029-016-0060-z)

Simulated Annealing Iterations

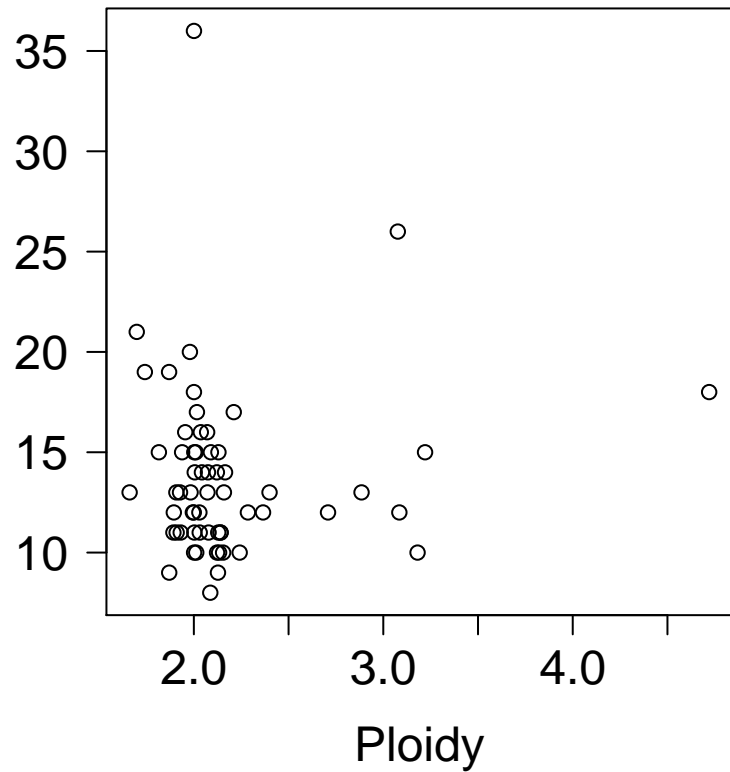

Simulated Annealing Iterations

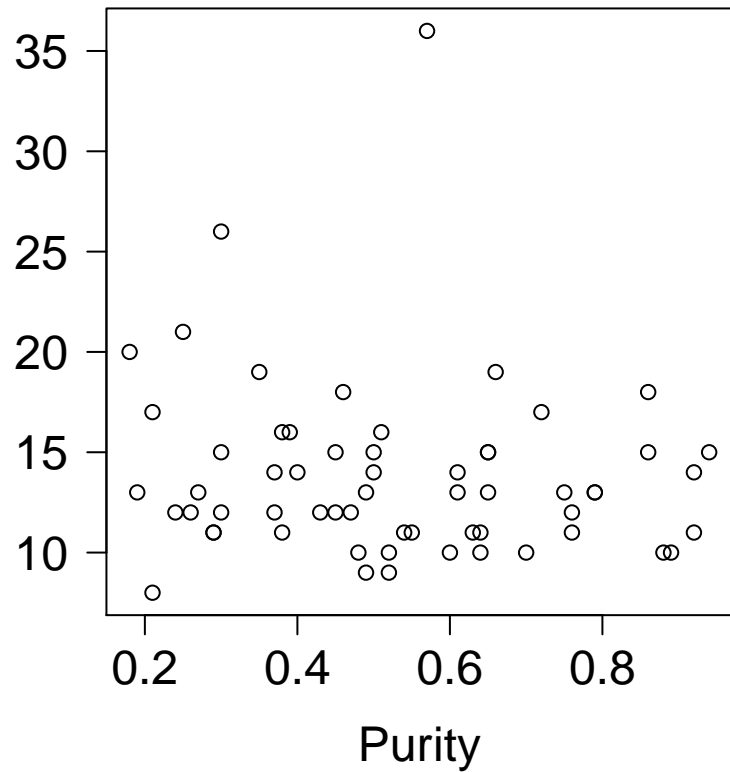

Supplement: Additional file 2: — Number of Simulated Annealing iterations. (PDF 5 kb) [file 13029_2016_60_MOESM2_ESM.pdf]
